# From Networking to Attitudinal Outcomes: Psychological Capital as a Mediator

**DOI:** 10.5334/pb.1264

**Published:** 2024-06-24

**Authors:** Rachel Huynen, Kathleen Bentein, Jessica Simon, Karen Valdiviezo, Audrey Babic

**Affiliations:** 1 Group and Organizational Psychology Department, University of Liege, 1, Quartier Agora Place des Orateurs (Bât.33), 4000 Liège, Belgium; 2 Department of Organization and Human Resources, School of Management (ESG), University of Quebec at Montreal (UQAM), C.P. 8888, Succursale Centre-ville, Montreal (QC) H3C 4R2, Canada; 3 Quantitative Psychology Department, University of Liege, 2, Quartier Agora Place des Orateurs (Bât.32), 4000 Liège, Belgium; 4 ARH, Organization, recruitment and training, University of Liege, 7, Place du 20-Août, 4000 Liège, Belgium

**Keywords:** networking, Psychological Capital, work satisfaction, work engagement, COR Theory

## Abstract

Building on conservation of resources (COR) theory and following recent recommendations, this study investigates the mediating role of Psychological Capital (PsyCap) in the relationships between networking behaviors and attitudinal outcomes (i.e., work engagement and work satisfaction). We propose that networking, as an investment of personal resources to gain access to other resources, contributes to the prediction of attitudinal outcomes. We surveyed 254 employees from a public Belgian administrative company. We use structural equation modelling and the bootstrapping method. PsyCap totally mediates the relationships between networking and both attitudinal outcomes. This study contributes to theoretical development by integrating Networking and PsyCap literatures into COR theory literature, and demonstrates the legitimacy of COR theory to explain these complex variables and their relationships.

## Introduction

In the past years, networking behaviors have received growing attention from organizational researchers. Networking refers to goal-directed behaviors performed to create, cultivate, and utilize interpersonal relationships (Gibson et al., 2014). Networking is positively linked with various work-related outcomes such as promotions, job performance or job search, and is recognized to play an important role in career success (for a review, see Gibson et al. 2014; Porter & Woo, 2015). Underlying this research stream is the idea that the burden of responsibility for one’s career has shifted from the organization to the individual, and that engaging in networking behaviors to build a set of interpersonal relationships is a specific strategy individuals can use to proactively manage their performance and promote their individual career (Hall, 1996; Hall & Moss, 1998).

While researchers have highlighted the long-term career related outcomes of networking, little is known about networking influence on attitudinal outcomes such as work satisfaction or work engagement. It is a shame as those attitudinal outcomes are more proximal than distal career outcomes and thus might be more salient for individuals. Moreover, research on networking has also largely overlooked the potential underlying mechanisms explaining the effects of networking behaviors on work outcomes. Indeed, as indicated by Gibson et al. (2014), research on networking has mainly focused on its antecedents and outcomes. In view of this, some scholars (Volmer & Wolff, 2018; Wingender & Wolff, 2023) recommend investigating potential mechanisms and processes that explain networking’s effects.

A possible underlying mechanism could be Psychological Capital (PsyCap), a core construct inspired from positive psychology (Seligman & Csikszentmihalyi, 2000), and encompassing four key personal resources: self-efficacy, optimism, hope, and resilience. The conservation of resources (COR) theory (Hobfoll, 1989, 1998; Hobfoll & Shirom, 2000) may be a useful theoretical framework to explain the psychological processes by which PsyCap could link networking and attitudinal outcomes (i.e., work satisfaction and work engagement). The basic tenet of COR theory is that even when stress is not occurring, people are motivated to retain, protect, and build resources (Hobfoll, 1989). Hobfoll (1989) defines resources as ‘those objects, personal characteristics, conditions, or energies that are valued by the individual or that serve as a means for attainment of these objects’ (Hobfoll, 1989, p. 516). COR theory also suggests that initial resource gain begets future gain, thus generating ‘gain spirals.’ These principles distinguish COR theory as a proactive rather than reactive theory. Therefore, we argue that networking can be conceptualized as a proactive investment of personal resources to gain access to contextual resources, which contributes to develop a caravan of key personal resources the PsyCap, which ultimately contributes to work engagement and work satisfaction.

By investigating how networking is linked to work engagement and work satisfaction through the mediating effect of PsyCap, the current study aims to contribute to the networking literature and PsyCap literature in several ways. First, as previously mentioned, while networking literature emphasizes career and performance outcomes (or production and behavioral outcomes in ten Brummelhuis & Bakker, 2012 classification of outcomes), little is known about the effect of networking on attitudinal outcomes. This study contributes to the exploration of the link between networking and attitudinal outcomes, namely work engagement and work satisfaction. Second, the present study intends to contribute to networking literature by showing that engaging in networking might not only contribute to the acquisition of contextual resources, that are located outside of the person, but might also play an important role in the development of a specific core personal resource, PsyCap, and that this caravan of key personal resources might play a central role in the prediction of attitudinal outcomes. Third, while studies regarding antecedents of PsyCap are mainly centered around the role of the external context (e.g., supportive organizational climate or specific interventions), and rarely explore the role of individual antecedents (Cenciotti et al., 2021), this study is the first to explore the role of an individual’s own networking behaviors in increasing PsyCap. Globally, as a side contribution, this study contributes to theoretical development by integrating networking and PsyCap literatures into COR theory literature, and should contribute to demonstrate the legitimacy of COR theory to explain these complex variables and their relationships.

## Literature Review

### Networking

Networking is defined by Wolff et al. (2011, p. 244) as “behaviors that are aimed at building, maintaining, and using informal relationships that possess the (potential) benefit of facilitating work-related activities of individual by voluntarily granting access to resources and maximizing common advantages.” The construct can be considered as a “*behavior syndrome*” (Frese et al., 1997), that is, a combination of interrelated behaviors consistently demonstrated by individuals. A distinctive characteristic of networking is its proactive nature. In comparison with seeking social support which refers to reactive behaviors used to solve a specific problem, networking refers to proactive behaviors that build the network of social contacts of individuals (Coleman, 1988). The concept of networking is also distinct from the concept of social capital. Networking is an individual level construct and focuses on individual behaviors, while social capital refers to a structural level concept and focuses on the quality and extent of existing relationship constellations (Wolff & Moser, 2009). Therefore, networking can be considered as an antecedent of social capital (Coleman, 1988).

Networking behaviors may be differentiated into internal and external ones, whether they occur inside or outside the organization (Michael & Yukl, 1993; Wolff & Moser, 2006). Examples of internal networking include going out for a drink with other workers after work or exchanging useful information with a colleague (Wolff et al., 2018). In contrast, external networking includes meeting clients or members of a professional association (McCallum et al., 2014). Past research showed that internal networking is more predictive of career and work satisfaction than external networking (Porter et al., 2016; Wolff & Moser, 2009). Moreover, according to Gibson et al. (2014), internal networking benefits may be better highlighted in noncompetitive organizations, characterized notably with lesser mobility across jobs and organizations, such as a public administrative company. With that in mind we decided to focus on internal networking in the present research. However, given that most research has studied networking behavior from a unitary perspective (McCallum et al, 2014), we will mostly review studies considering networking behavior as a unitary construct.

In the literature, networking is considered as an important strategy in an individual’s work and career because it represents an investment of resources to obtain access to other resources that facilitate individual effectiveness (Porter & Woo, 2015). Based on the COR theory perspective (Hobfoll, 2002; ten Brummelhuis & Bakker, 2012), networking has been conceptualized as an investment of two types of personal resources: structural constructive resources, and transient energy resources (Volmer & Wolff, 2018). Because it entails the usage of important skills that represents durable assets for the person, networking includes the investment of structural constructive resources. And, because people also invest volatile resources, like time, effort or self-control, which once they are used, cannot be re-used for other purposes, networking also encompasses transient energy resources. Wingender and Wolff (2023) recently empirically demonstrated that engaging in networking behaviors includes the investment of transient energy resources as it depletes self-control resources. These two types of personal resources are used in order to gain access to contextual resources that are located outside of the self (e.g., task advice, strategic information). Wolff et al. (2008) further distinguish between proximal contextual resources obtained from a single personal networking contact (i.e., social support), and distal contextual resources obtained from an entire network of contact (i.e., career success or power that people accrue from their network) and which can be seen as an accumulation of proximal contextual resources. In the present study, we further propose that networking, as a proactive investment of personal resources, might not only contribute to the acquisition of contextual resources but might also play an important role in the development of a specific core personal resource, the PsyCap.

### Psychological Capital

PsyCap refers to “*an individual’s positive psychological state of development and is characterized by: (1) having confidence (self-efficacy) to take on and put in the necessary effort to succeed at challenging tasks; (2) making a positive attribution (optimism) about succeeding now and in the future; (3) persevering toward goals and, when necessary, redirecting paths to goals (hope) in order to succeed; and (4) when beset by problems and adversity, sustaining and bouncing back and even beyond (resiliency) to attain success*.” (Luthans, 2007, p. 3). PsyCap is thus a higher-order construct that represents the commonality among self-efficacy, optimism, hope, and resiliency. While previous literature has demonstrated that these four positive variables are conceptually and psychometrically distinct, research has shown that the global construct of PsyCap has a stronger effect on work outcomes than its four underlying components taken separately (Avey et al. 2011; Luthans et al., 2007). For example, the relationship between PsyCap and job performance was stronger than the relationships between each of its components considered individually, and job performance (Luthans et al., 2005).

An important characteristic of PsyCap is its state-like nature, meaning that it is malleable and open to change and development (e.g., Luthans et al., 2007). PsyCap is not like personality or core self-evaluation traits, which are relatively fixed, but can be changed by experience and developed in training (e.g., Luthans et al., 2007).

Numerous studies have demonstrated PsyCap’s positive influence on various work outcomes, both at the individual and organizational level. For example, PsyCap increases job performance, work engagement, positive organizational citizenship behaviors (OCB), and also reduces stress and undesirable work behaviors such as cynicism or turnover intentions (e.g., Newman et al., 2014; Nolzen, 2018).

From the COR theory perspective, PsyCap might be seen as a caravan of key personal resources (Hobfoll, 2002, 2011). First, PsyCap represents management resources that facilitates the mobilization of other resources (Thoits, 1994), and as such might be considered as key resources (ten Brummelhuis & Bakker, 2012). Second, based on Hobfoll’s (2011) argument that resources do not exist individually but rather travel in packs or “caravans,” PsyCap, as a combination of four psychological key resources, can be considered as a resource caravan (Hobfoll, 2011).

### PsyCap as a mediator of the relationship between networking and attitudinal outcomes

Although researchers have shown that networking is positively associated with numerous production or behavioral outcomes, like work performance, career management and success, or job search (Gibson et al., 2014; Porter & Woo, 2015), little is known about networking’s impact on attitudinal outcomes. Drawing on COR theory, we propose that networking, as an investment of personal resources to gain access to other resources, also contributes to the prediction of attitudinal outcomes, like work engagement (defined as “*a positive, fulfilling, affective emotional state of work-related well-being;*” Bakker et al., 2008, p. 188) and work satisfaction (defined as *“a pleasurable or positive emotional state resulting from the appraisal of one’s job or job experiences;”* Locke, 1976, p. 1300). Because these two attitudinal outcomes refer to positive attitudes resulting from an individual’s evaluation of work-related well-being or of job experiences, they might be seen as attitudinal outcomes of resource acquisition process (ten Brummelhuis & Bakker, 2012; Volmer & Wolff, 2018). A few empirical studies support the relationship between networking and both work engagement and work satisfaction. Nigah et al. (2012) found that satisfaction with buddying (i.e., a particular mechanism of newcomers’ socialization close to mentoring) was positively related to work engagement, in a sample of 78 newcomers in the professional service sector. Volmer and Wolff (2018) found that daily networking at work was positively related to daily employees’ job satisfaction, in a sample of academic employees. Porter et al. (2016) found that internal networking was positively associated with job satisfaction.

In accordance with COR theory (Hobfoll, 2002, 2011), we further propose that PsyCap, as a caravan of key personal resources, might play a mediating role in the relationship between networking and those two attitudinal outcomes. Our hypothesis directly aligns with the principles of resources investment and gain spiral. Employees are required to invest resources to gain additional ones (Hobfoll, 2011). We take this concept a step further and argue that networking behaviors constitute an investment of resources that in itself helps to develop personal resources. Given its proactive nature in acquiring valuable resources or the potential to receive them in the future, networking can be perceived as a positive experience leading to the development of PsyCap which represents a form of self-beliefs regarding future resource availability (e.g., self-efficacy, optimism, hope, and resiliency). Several studies on proactive behaviors and proactivity indirectly support this connection between active behaviors aimed at changing or coping with the environment (e.g., networking behaviors) and a future-oriented positive mindset (Cai et al., 2015; Santilli et al., 2016). Recently, Volmer and colleagues (Volmer & Wolff 2018; Volmer et al., 2021) found that daily networking was positively associated with daily career optimism and career optimism, malleable states that are closed to the optimism component of the PsyCap. Another argument for this reasoning could be found in the Socialization Resources Theory (SRT). According to Saks and Gruman (2011), through the process of socialization, or the process by which an individual acquires the knowledge and values that are essential to adjust to his or her new role, newcomers develop their PsyCap which in turn increases socialization outcomes such as work organizational commitment, work satisfaction or job performance. Furthermore, research suggests that the acquisition of contextual resources such as social support enhances key personal resources of PsyCap. For example, a recent study conducted by Wang & Lei (2023) demonstrated that proactive individuals who receive higher levels of social support experience increased feelings of hope, ultimately increasing their job satisfaction. In the same vein, Wolter et al. (2019) found that self-efficacy partially mediated the relationship between social support and work engagement among German police officers. Additionally, a recent systematic review conducted by Galanis et al. (2022) showed that social support improves resilience among nurses. Finally, a study on Turkish teacher candidates found that perceived social support predicts optimism (Tras et al., 2021).

PsyCap, considering its positive nature, will promote a positive interpretation of the social environment (i.e., satisfaction), and, as an agentic mindset that is oriented to produce the desired external conditions (Cenciotti et al., 2021), will promote work engagement. Previous research showed that individuals high on PsyCap reported higher levels of work engagement (e.g., Paek et al., 2015; Simons & Buitendach, 2013). A longitudinal study on white-collar employees showed that increases in PsyCap predicted increases in work engagement over time (Alessandri et al., 2018). The positive association between PsyCap and work engagement was also highlighted in Loghman et al. (2023) recent meta-analysis comprising 254 independent samples and more than 96,000 participants. In the same vein, previous research found a positive relationship between PsyCap and work satisfaction (e.g., Abbas et al., 2014; Luthans et al., 2007). Recent meta-analyses confirmed PsyCap’s positive influence on work satisfaction (Avey et al. 2011; Loghman et al., 2023). So, by providing nurturing contextual resources (e.g., social support, advice, etc.), networking allow workers to develop their key personal resources of self-efficacy, optimism, hope, and resilience, or PsyCap, which would broadly facilitate the attainment of other resources, and have a positive influence on work engagement and work satisfaction.

We are only aware of one study that investigated the relationship between networking and PsyCap. This study, conducted by Kauffeld and Spurk (2022) on academic scientists, tested the mediating role of networking in the relationship between PsyCap and career success. The authors assumed that high PsyCap individuals should be more prone to engage in networking and subsequently use their network contacts to help them in their career. Although Kauffeld and Spurk found a positive bivariate correlation between PsyCap and internal networking, they did not find evidence of a positive indirect effect of PsyCap on career success via internal networking. Based on this non-significant result and on the aforementioned reasoning, we maintain that, even if networking and PsyCap may reciprocally influence one another, internal networking is likely to exhibit a stronger predictive relationship with PsyCap (as opposed to the reverse causal ordering).

Therefore, in line with this reasoning, we hypothesize that networking will have a positive impact on work engagement and work satisfaction, through its positive impact on PsyCap. Thus, we postulate that:

*Hypothesis 1*: PsyCap mediates the relationship between internal networking and work satisfaction.

*Hypothesis 2*: PsyCap mediates the relationship between internal networking and work engagement.

**Figure 1:**
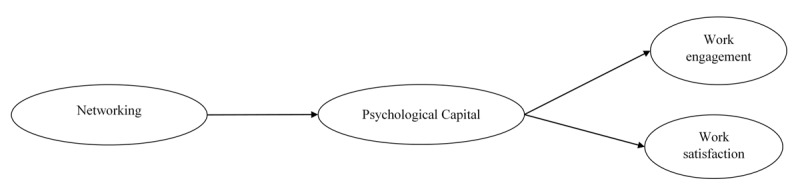
Hypothesized theoretical model.

Figure 1 depicts the hypothesized theoretical model. As shown in this figure, PsyCap is assumed to mediate the relationships between internal networking and attitudinal outcomes (i.e., work satisfaction and work engagement).

## Materials and Method

Prior to launching the survey, the present study and its design were presented for approval to the ethical committee of the faculty of psychology of the researchers’ university. The final decision of the ethical committee was positive suggesting that the present study fulfills all the ethical rules regarding the methodological design.

### Sample and Procedure

This study was conducted in a public company of a Belgian federal administrative service. The well-being service of the company sent an email to employees explaining the study and containing the link to the online survey. One thousand three hundred ninety-eight French-speaking employees were invited to participate in this study. Participants were given three weeks to complete the survey. Two weeks after the launch of the survey, each employee received a second email reminding them of the end-date and thanking the participants.

We received a total of 254 responses, which represents a participation rate of 18%. Participants were 155 women and 99 men. Seven percent of the participants had 25 years old or less, 24% were between 26 and 35 years old, 32% were between 36 and 45 years old, 18% were between 46 and 55 years old and finally 19% had 55 years old or more. Forty-seven percent of the participants occupied an administrative position, thirty-four a specific position, and seventeen percent a technical position. The majority (37%) of the respondents had between 6 and 15 years of tenure in the company and had a full-time job (86%).

### Measures

*Internal networking* was measured using the internal networking dimension of Wolff and Moser’s networking scale (Wolff & Moser, 2006) which measures to what extent participants carry out behaviors aimed at developing one’s internal network within the organization. This dimension is composed of three types of networking; building internal contacts (6 items; e.g., “I use company events to make new contacts”), maintaining internal contacts (7 items; e.g., “I catch up with colleagues from other departments about what they are working on”) and using internal contacts (8 items; e.g., “I use my contacts with colleagues in other departments in order to get confidential advice in business matters”). As participants speak French, the original English internal networking questionnaire was translated following a translation back-translation procedure (Brislin, 1970). There was no major discrepancy between the original and translated versions, so the translation process was considered appropriate. People responded on a 4-point Likert-type scale (from 1 = never to 4 = always). A confirmatory factor analysis (CFA) was performed on a three-factor model (Hu & Liden, 2011). The results of the CFA indicate that these three factors are distinct and fall under a second-order Networking behaviors construct, (χ^2^(206) = 556.56, p < .001, RMSEA = .08, SRMR = .06, CFI = .91). The factor loading range of the three dimensions onto Networking construct is .76 to .85. Therefore, overall Networking was used as a latent factor (α = .89) in the subsequent analyses.

*PsyCap* was measured with the French version of the Psychological Capital Questionnaire (Luthans et al., 2007). This scale consists of 24 items equally divided in four subscales: hope (e.g., “I can think of many ways to reach my current work goals”), resilience (e.g., “I can get through difficult times at work because I’ve experienced difficulty before”), self-efficacy (e.g., “I feel confident analyzing a long-term problem to find a solution”) and optimism (e.g., “When things are uncertain for me at work, I usually expect the best”). Each item is answered using a 6-point Likert-type scale (from 1 = strongly disagree to 6 = strongly agree). The results of the CFA indicate that four factors are distinct and fall under a second-order PsyCap construct, (χ^2^(248) = 710.19, p < .001, RMSEA = .08, SRMR = .06, CFI = .92). The factor loading range of the four dimensions onto the PsyCap construct is .79 to .91. Therefore, PsyCap was used as a latent factor (α = .91) in the subsequent analyses.

*Work engagement* was assessed using the validated French short version of the Utrecht Work Engagement Scale (UWES) (Schaufeli et al., 2006). The scale includes three dimensions: vigor (three items; e.g. “At my work, I feel bursting of energy”), dedication (three items; e.g. “I am enthusiastic about my job”) and absorption (three items; e.g. “I feel happy when I am working intensely”). The response scale ranged from 0 (never) to 6 (always). The results of the CFA indicate that three factors are distinct and fall under a second-order Work engagement construct, (χ^2^(24) = 116.36, p < .001, RMSEA = .08, SRMR = .07, CFI = .94). The factor loading range of the three dimensions onto Work engagement construct is .82 to .89. Therefore, Work engagement behaviors was used as a latent factor (α = .91) in the subsequent analyses.

*Work satisfaction* was measured with the Michigan Organizational Assessment Questionnaire (MOAQ-3) (Cammann et al., 1983). This scale consists of three items (“In general, I like working here,” “All in all, I am satisfied with my job,” and “In general, I don’t like my job”), answered using a 6-point Likert-type scale ranging from (1) strongly disagree to (6) strongly agree. Cronbach’s alpha was .85.

Several socio-demographic variables (i.e., gender, age, organizational tenure, working time) were considered as potential control variables. Consistent with the full partial method (Little, 2013), we pointed out that, after removing the nonsignificant effects, two socio-demographic variables were significantly related to the constructs of our model: age was significantly positively related to PsyCap; working time was significantly negatively related to work engagement and work satisfaction. Consequently, in our analyses, we included these two socio-demographic variables as covariates to control for their effects.

### Data analysis

We used items as indicators only for work satisfaction. For others constructs, we averaged items by dimension to create indicators representing the dimensions (i.e., Internal networking was represented by three indicators; PsyCap by four indicators; Work engagement by three indicators). Structural equation modeling analyses (SEM) were performed using Mplus 6 (Muthèn & Muthèn, 2010). We analyzed data following a two-stage process (Anderson & Gerbing, 1988). Firstly, we assessed the measurement model through confirmatory factor analyses to evaluate the independence of the constructs examined in our study. Second, we proceeded with the assessment of the hypothesized structural relationships among latent variables. We also used the bootstrapping technique to estimate indirect effects (Preacher & Hayes, 2008).

## Results

### Confirmatory factor analyses

Firstly, we examined the fit of our hypothesized four-factor measurement model (i.e. Internal networking, PsyCap, Work engagement and Work satisfaction). Results indicate that this four-factor measurement model fit the data reasonably well (χ^2^(59) = 157.70, p < .001, SRMR = .05, RMSEA = .08, CFI = .95). Starting from this four-factor model, we tested four more constrained measurement models to ensure that our constructs were independent (Anderson & Gerbing, 1988), (1) a first three-factor model (Work engagement and Work satisfaction = 1 factor), (2) a second three-factor model (PsyCap and Work engagement = 1 factor), (3) a third three-factor model (PsyCap and Work satisfaction = 1 factor), and (4) a one-factor model (all the variables as a single-factor). Chi-square difference tests were then used to compare the fit of each of these nested models with that of the four-factor model (Bentler & Bonett, 1980). The significance of the chi-square differences suggests that the four-factor model was superior to the other compared models. Consequently, we treated these four constructs as independent from each other in subsequent analyses. Table 1 displays fit indices of these alternative models.

**Table 1:** Fit indices for measurement models.


MODEL	χ^2^	*DF*	χ^2^/*DF*	CFI	RMSEA	SRMR	Δχ^2^ (ΔDF)

4-factor model	157.70	59	2.67	.95	.08	.05	---

3-factor model (1) : Work engagement with Work satisfaction	214.78	62	3.46	.92	.10	.06	57.08(3)***

3-factor model (2) : Psychological Capital with Work satisfaction	249.23	62	4.02	.90	.11	.07	91.53(3)***

3-factor model (3): Psychological Capital with Work engagement	275.54	62	4.44	.89	.12	.07	117.84(3)***

1-factor model	508.42	65	7.82	.77	.16	.10	350.72(6)***


*Note. N* = 254. χ^2^ = Minimum Fit Function Chi-Square; df = degrees of freedom; CFI = Comparative Fit Index; RMSEA = root-mean-square error of approximation; SRMR = standardized root mean square residual; Δχ^2^ = chi-square difference tests. ***p < .001.

### Relationships among variables

Means, standard deviations, Cronbach’s alphas and correlations among variables are presented in Table 2. Internal consistency reliabilities ranged from .85 to .91. As expected, internal networking behaviors are significantly positively correlated to PsyCap (r = .40, p < .001), work engagement (r = .35, p < .001) and work satisfaction (r = .26, p < .001). PsyCap is also significantly positively correlated to work engagement (r = .61, p < .001) and work satisfaction (r = .70, p < .001).

**Table 2:** Descriptive statistics and inter-correlations among variables.


	VARIABLES	*M*	*SD*	1	2	3	4	5	6

1	Age	---	---	---					

2	Working time	---	---	.10	---				

3	Internal networking	2.42	.49	–.03	–.09	(.89)			

4	Psychological Capital	3.97	.49	.12*	–.10	.40***	(.91)		

5	Work engagement	5.10	.99	.10	–.17**	.35***	.61***	(.91)	

6	Work satisfaction	4.81	1.03	.06	–.22**	.26***	.70***	.76***	(.85)


*Note. N* = 254. Correlations among variables are provided below the diagonal and Cronbach’s alphas are provided on the diagonal. Absence of means and standard deviations for gender and organizational tenure because the answers were beforehand categorized in the questionnaire. *p < .05., ***p < .001.

Table 3 presents fit indices for the hypothesized structural model (Model 1) and one alternative model (Model 2). Model 1 fitted the data reasonably well, as indicated by the following indices: χ^2^ (84) = 206.30, *p* < .001, RMSEA = .08, SRMR = .06, CFI = .94. Starting from Model 1, we added paths from Internal networking to Work engagement and Work satisfaction (Model 2) (χ^2^ (82) = 202.42, *p* < .001, RMSEA = .08, SRMR = .06, CFI = .94), because these direct paths were theoretically plausible, and because it helps us to extend our understanding of the mediation process (MacKinnon et al., 2004). The Chi-square difference test was used to compare the fit of Model 2 (partial mediation model) with that of Model 1 (full mediation model; Bentler & Bonett, 1980). Results indicate that Model 2 was not significantly superior to Model 1, and suggest that the links between Internal networking and Work engagement and Work satisfaction are being fully mediated by PsyCap. We thus retained Model 1 as the best fitting model.

**Table 3:** Fit indices for structural models.


MODEL	χ^2^	*DF*	χ^2^/*DF*	CFI	RMSEA	SRMR	COMPARISON	Δχ^2^ (ΔDF)

Model 1: Hypothesized theoretical model	206.30	84	2.46	.94	.08	.06	---	---

Model 2 : Model 1 + Paths between Internal networking, Work engagement and Work satisfaction	202.42	82	2.35	.94	.08	.06	1 VS 2	3.88(2)


*Note. N* = 254. χ^2^ = Minimum Fit Function Chi-Square; df = degrees of freedom; CFI = Comparative Fit Index; RMSEA = root-mean-square error of approximation; SRMR = standardized root mean square residual; Δχ^2^ = chi-square difference tests. ***p < .001.

**Figure 2:**
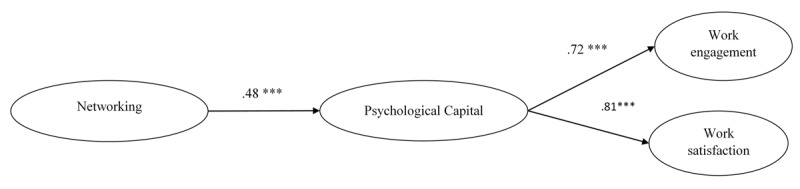
Completely standardized path coefficients for the retained model (model 1). For the sake of clarity, only structural relationships are shown. ***p < .001.

Standardized parameter estimates for this model are shown in Figure 2. For ease of presentation, we show the structural model rather than the full measurement model. Internal networking behaviors were positively associated with PsyCap (β = .48, p < .001), which, in turn, was positively related to Work engagement (β = .72, p < .001) and Work satisfaction (β = .81, p < .001). This model accounted for 25% of the variance of PsyCap, 53% of the variance of Work Engagement and 66% of the variance of Work Satisfaction.

Results of the bootstrap analyses, presented in Table 4, indicated that the indirect effect of Internal networking on Work Satisfaction through PsyCap was significant (indirect effect = .39; BCa 95% CI = [.30; .47]), supporting our Hypothesis 1. In the same vein, the indirect effect of Internal networking on Work Engagement through PsyCap was also significant (indirect effect = .35; BCa 95% CI = [.25; .44]), supporting our Hypothesis 2. Thus, PsyCap totally mediated the effects of Internal networking on Work satisfaction and Work engagement.

**Table 4:** Indirect pathways using bootstrapping.


INDIRECT EFFECT : X →M → Y	BOOTSTRAPPING		PERCENTILE 95% CI	
	
	EFFECT	SE	LOWER	UPPER

Internal networking → Psychological Capital → Work engagement	.347	.057	.253	.440

Internal networking → Psychological Capital → Work satisfaction	.386	.053	.300	.473


*Note*. *N* = 254. SE = Standard Error; CI = Confidence Interval; 10,000 bootstrap samples.

## Discussion

Using COR theory (Hobfoll, 1989, 1998) as a framework to build our conceptual model, the present study was designed to advance our understanding of the relationship between networking and two attitudinal outcomes, work engagement and work satisfaction, through the mediating effect of PsyCap. We observed solid support for our hypothesized model, as both the indirect effect of internal networking on work engagement and on work satisfaction through PsyCap were significant.

Our study makes significant contributions to the networking literature. First, we found strong evidence for a positive relationship between networking and two attitudinal outcomes, namely work engagement and work satisfaction. While previous literature mainly associated networking with production and behavioral outcomes (Gibson et al., 2014; Porter & Woo, 2015), the present study contributes to recent literature that has begun to explore how engaging in networking contributes to attitudinal outcomes. This finding is particularly important because, even if the goal of networking behaviors is to obtain resources that are tied to performance and career outcomes, networking might help individuals to develop positive attitudes at work.

Second, our results demonstrate that networking is a means to build PsyCap. This knowledge expands existing networking theory in showing that engaging in networking is not only associated with contextual resources, that are located outside of the self, but also with a caravan of key personal resources, PsyCap. This notion that accumulation of one type of resource facilitates the accumulation of another type of resource is already theoretically developed in the career literature. Hirschi (2012) developed an integrated resource model, where four different types of resources, including social resources and psychological resources, are proposed to work in tandem in promoting successful career development. In their model, social capital resources (e.g. networks) might enhance psychological resources (e.g., PsyCap), among other things by providing role models and psychological support. Our study is one of the first to empirically demonstrate the specific link between networking and PsyCap.

Third, we further found that this key resource variable, PsyCap fully mediated the association between networking and attitudinal outcomes. This finding extends networking research by providing further insights into how networking leads to positive attitudinal outcomes. Along with COR theory, which suggests that resources should stimulate an increase of further resources and valuable outcomes, this mediation mechanism might be conceived as part of a gain spiral process. We argue that it is because its proactive nature that engaging in networking activities to acquire valuable resources or the potential to receive them in the future contributes to the development of PsyCap which represents a form of self-beliefs regarding future resource availability (e.g., self-efficacy, optimism, hope, and resiliency). It is because the employee engages in networking activities aimed at adapting to or modifying his/her environment, or an *active* investment of personal resources to acquire contextual resources, that he/she develops beliefs about forthcoming resource availability and a future-oriented positive mindset (Cai et al., 2015; Santilli et al., 2016). This finding is in line with two recent studies showing that daily investments in networking positively relates to work-non-work enrichment, job satisfaction, career optimism, or career satisfaction, through a personal affective resource, positive affect at work (Baumeler et al., 2018; Volmer & Wolff, 2018).

Our study also expands our knowledge of the individual antecedents of PsyCap. Given the increased interest of researchers and practitioners in promoting employees’ PsyCap (e.g., Lupșa et al., 2020; Wu & Nguyen, 2019) and the relative paucity of studies exploring individual antecedents, recognizing the role of networking in reinforcing employees’ PsyCap is an important finding. Even if PsyCap is explicitly defined as trainable and open to development (Luthans et al., 2007), knowing that it could be developed through proactive behaviors initiated by employees themselves, is of primary importance in the actual turbulent context of work. This finding is in line with Vogt et al. (2016) who found that job crafting, defined as self-initiated changes regarding job resources (and job demands), helps to build PsyCap.

Moreover, our results confirm the existence of strong links between PsyCap and positive attitudinal outcomes. The relationship between PsyCap and work engagement is similar to what was obtained in previous studies (e.g., Nigah et al., 2012: r = .61). On the other hand, for work satisfaction, if our results contrast with weaker correlations found in past research (e.g., Avey et al., 2011: r = 54; Abbas et al., 2014: r = .49), they are in line with those obtained by Loghman et al. (2023) in their meta-analysis (work satisfaction: r = .68; work engagement: r = .71), suggesting that past work has tend to underestimate these links. In line with COR Theory, this suggests that PsyCap resources act as a strong driver to foster desirable employee attitudes. Indeed, as individuals “*higher in PsyCap expect good things to happen at work (optimism), believe they create their own success (efficacy and hope), and are more impervious to setbacks (resilience)*” they are more prone to develop positive attitudes towards their work (Avey et al. 2011, p 132).

In line with Volmer and Wolff (2018), we integrate networking and PsyCap literature into COR theory literature, because it provides a suitable framework to organize resources, that are at the heart of networking and PsyCap concepts. We conceptualize networking as an investment of two types of personal resources, structural constructive resources (i.e., skills) and transient energy resources (i.e., time, effort, self-control), to gain access to contextual resources. Through engaging in resource’s investment process, people develop a caravan of key personal resources (i.e., self-efficacy, optimism, hope, and resiliency), the PsyCap, that will promote a positive interpretation of the social environment (i.e., work satisfaction), and an orientation to produce the desired external conditions (i.e., work engagement). Our findings tend to support these theoretical propositions, but future research might want to explore them in finer-grained details, for example, by measuring the cycles of investment of precise personal resources and the gain of specific contextual and personal resources over short periods of time.

### Limitations and future perspectives

The present study has several limitations that need to be acknowledged. First, its cross-sectional design prevents making strong causal inferences on the investigated relationships (Kline, 2011). We thus recommend future research to replicate our study using longitudinal or experimental designs (see Wingender & Wolff, 2023 for an example of experimental laboratory studies).

Second, data are based on self-reported measures. Because our research focused on workers’ perceptions, we needed to obtain self-perceptions regarding these constructs. However, using self-reported measures may have reduced the validity of our results due to two important biases, i.e., the social desirability influence bias (Grimm, 2010) and the common method bias (Podsakoff et al., 2012). Nevertheless, in order to counter such bias, we followed several scholars’ recommendations (e.g., Podsakoff et al., 2012) and took several precautions on both methodological and statistical levels. On one hand, at the methodological level, participants were assured that their responses were anonym and confidential and that there were no right or wrong answers to the questions. We also used largely validated questionnaires in the literature, and performed confirmatory factor analyses to demonstrate their validity. On the other hand, at the statistical level, we conducted the Harman’s one-factor test and results indicated that the common method bias was not a major threat to our results.

Third, our study didn’t include a measure of external networking. However, external and internal networking are likely to play distinct roles in the processes by which they influence attitudinal outcomes. Porter et al. (2016) show, for example, that the likelihood of voluntary turnover was negatively predicted by internal networking through job embeddedness and positively predicted by external networking through job offers, representing opportunities to leave. So, each type of networking behavior might promote access to different interpersonal resources, and through distinct mechanisms. To have a better grasp of these differential resources and processes, future studies should measure both internal and external networking behaviors. Moreover, beyond the classification of external versus internal networking, there are other networking behaviors that are of interest. Indeed, with the advent of technology and social media, new types of networking behaviors have emerged, such as the use of professional social networking site (SNS) (e.g., LinkedIn) (e.g., Pena et al., 2022). Although research suggests that the use of SNS yields the same benefits as face to face networking (Baumann & Utz, 2021), we still don’t know much about the processes underlying these benefits (Anderl et al., 2023). Therefore, given the growing importance of these behaviors and the paucity of the literature available, future studies should include a measure of these behaviors as well.

Finally, another limitation concerns the generalizability of our results. As our sample included only workers from an administrative public company, findings may not be applicable to populations of workers within private companies. It is possible that culture or some work characteristics or the profile of workers will differ in companies coming from the private sector. Indeed, through a large Belgium survey, Buelens and Van den Broeck (2007) found that public sector employees were, for example, less motivated by challenge and personal growth compared to private sector employees. Other studies also found that public sector employees showed weaker internal work motivation than their private sector counterparts (Aryee, 1992). This situation might have decreased the mean observed, in our sample, for networking behaviors, but also reduced the variance of reported employees’ networking behaviors. As it is harder to detect relationships between variables when dealing with variables with low variance, our results might represent a conservative estimate of the relationships between networking behaviors and other variables of the model. These elements might also lead public and private employees to adopt different kinds of networking behaviors. Therefore, future research should replicate our model with other samples. Further, as this study was conducted in Belgium, it would be interesting to investigate how networking behaviors may influence work satisfaction and work engagement through PsyCap in other cultural contexts.

### Practical implications

The results of this study emphasize the importance of internal networking for promoting employee positive attitudes toward their work. Specifically, we provide insights into how internal networking may foster employee psychological resources and subsequently increase their work satisfaction and work engagement. In the actual labor context, keeping employees engaged and satisfied at work is essential and benefits not only employees but also organizations. Indeed, as the research shows, work satisfaction and work engagement are linked to various positive outcomes such as job performance, OCB, lower turnover intentions, and workplace incivility (e.g., Judge et al., 2020).

Organizations and HR managers should therefore adopt and strengthen efforts to encourage networking among employees. In line with the three types of internal networking, this can be done by prompting and training employees to build internal contacts, to use and maintain them. This includes for example organizing regular company events and rewarding attendance, providing networking training or workshops, encouraging collaboration among different departments, or favoring environments where employees can easily interact (McCallum et al., 2014). Indeed, fostering networking among employees may also be done by the appropriate structuration of the work environment, which can promote daily networking such as informal meetings at the coffee machine (Volmer & Wolff, 2018). Finally, we recommend organizations and HR managers to also include networking training in the socialization process in an effort to raise newcomers’ awareness and understanding of networking benefits.

As our results demonstrated PsyCap central role in building work satisfaction and work engagement and given its openness to development, we also encourage organizations and HR managers to implement training interventions aimed at developing workers’ PsyCap. PsyCap can be developed through Luthans and colleagues’ PsyCap Intervention (PCI) model. Guidelines for the PCI and further information on how to build and sustain PsyCap can be found in the substantial literature available (e.g., Luthans et al., 2010).

## Data Accessibility Statement

The datasets generated during and/or analyzed during the current study are available from the corresponding author on reasonable request.

## References

[1] Abbas, M., Raja, U., Darr, W., & Bouckenooghe, D. (2014). Combined effects of perceived politics and psychological capital on job satisfaction, turnover intentions, and performance. Journal of Management, 40(7), 1813–1830. DOI: 10.1177/0149206312455243

[2] Alessandri, G., Consiglio, C., Luthans, F., & Borgogni, L. (2018). Testing a dynamic model of the impact of psychological capital on work engagement and job performance. Career Development International, 23(1), 33–47. DOI: 10.1108/CDI-11-2016-0210

[3] Anderl, C., Baumann, L., & Utz, S. (2023). Social networking site use in professional contexts. In J. Skopek (Ed.), Research Handbook on Digital Sociology (pp. 179–194). Edward Elgar Publishing. DOI: 10.4337/9781789906769.00018

[4] Anderson, J. C., & Gerbing, D. W. (1988). Structural equation modeling in practice: A review and recommended two-step approach. Psychological Bulletin, 103(3), 411–423. DOI: 10.1037/0033-2909.103.3.411

[5] Aryee, S. (1992). Public and private sector professionals: A comparative study of their perceived work experience. Group and Organization Management, 17(1), 72–85. DOI: 10.1177/1059601192171006

[6] Avey, J. B., Reichard, R. J., Luthans, F., & Mhatre, K. H. (2011). Meta-analysis of the impact of positive psychological capital on employee attitudes, behaviors, and performance. Human Resource Development Quarterly, 22(2), 127–152. DOI: 10.1002/hrdq.20070

[7] Bakker, A. B., Schaufeli, W. B., Leiter, M. P., & Taris, T. W. (2008). Work engagement: An emerging concept in occupational health psychology. Work & Stress, 22(3), 187–200. DOI: 10.1080/02678370802393649 20103890

[8] Baumann, L., & Utz, S. (2021). Professional networking: Exploring differences between offline and online networking. Cyberpsychology: Journal of Psychosocial Research on Cyberspace, 15(1). DOI: 10.5817/CP2021-1-2

[9] Baumeler, F., Johnston, C. S., Hirschi, A., & Spurk, D. (2018). Networking as predictor of work-nonwork enrichment: Mechanisms on the within-and between-person level. Journal of vocational behavior, 109, 166–177. DOI: 10.1016/j.jvb.2018.10.015

[10] Bentler, P. M., & Bonett, D. G. (1980). Significance tests and goodness of fit in the analysis of covariance structures. Psychological bulletin, 88(3), 588–606. DOI: 10.1037/0033-2909.88.3.588

[11] Brislin R. W. (1970). Back translation for cross-cultural research. Journal of Cross-Cultural Psychology, 1(3), 185–216. DOI: 10.1177/135910457000100301

[12] Buelens, M., & Van den Broeck, H. (2007). An analysis of differences in work motivation between public and private sector organizations. Public Administration Review, 67(1), 65–74. DOI: 10.1111/j.1540-6210.2006.00697.x

[13] Cai, Z., Guan, Y., Li, H., Shi, W., Guo, K., Liu, Y., … & Hua, H. (2015). Self-esteem and proactive personality as predictors of future work self and career adaptability: An examination of mediating and moderating processes. Journal of Vocational behavior, 86, 86–94. DOI: 10.1016/j.jvb.2014.10.004

[14] Cammann, C., Fichman, M., Jenkins, G. D., & Klesh, J. (1983). Michigan organizational assessment questionnaire. In S. E. Seashore, E. E. Lawler, P. H. Mirvis, & C. Cammann (Eds.), Assessing organizational change: A guide to methods, measures, and practices (pp. 71–138). New York: Wiley-Interscience.

[15] Cenciotti, R., Alessandri, G., Borgogni, L., & Consiglio, C. (2021). Agentic capabilities as predictors of psychological capital, job performance, and social capital over time. International Journal of Selection and Assessment, 30(2), 249–264. DOI: 10.1111/ijsa.12357

[16] Coleman, J. S. (1988). Social capital in the creation of human capital. American Journal of Sociology, 94, S95–S120. DOI: 10.1086/228943

[17] Frese, M., Fay, D., Hilburger, T., Leng, K., & Tag, A. (1997). The concept of personal initiative: Operationalization, reliability and validity in two German samples. Journal of Occupational and Organizational Psychology, 70(2), 139–161. DOI: 10.1111/j.2044-8325.1997.tb00639.x

[18] Galanis, P., Katsiroumpa, A., Vraka, I., Siskou, O., Konstantakopoulou, O., Katsoulas, T., & Kaitelidou, D. (2022). Relationship between social support and resilience among nurses: A systematic review. International Journal of Caring Sciences, 15(3), 1675–1681. DOI: 10.1101/2022.09.04.22279592

[19] Gibson, C., Hardy, J. H., & Buckley, M. R. (2014). Understanding the role of networking in organizations. Career Development International, 19(2), 146–161. DOI: 10.1108/CDI-09-2013-0111

[20] Grimm, P. (2010). Social desirability bias. Wiley International Encyclopaedia of Marketing. Chichester: Wiley. DOI: 10.1002/9781444316568.wiem02057

[21] Hall, D. T. (1996). Protean careers of the 21st century. Academy of management perspectives, 10(4), 8–16. DOI: 10.5465/ame.1996.3145315

[22] Hall, D. T., & Moss, J. E. (1998). The new protean career contract: Helping organizations and employees adapt. Organizational dynamics, 26(3), 22–37. DOI: 10.1016/S0090-2616(98)90012-2

[23] Hirschi, A. (2012). The career resources model: An integrative framework for career counsellors. British Journal of Guidance & Counselling, 40(4), 369–383. DOI: 10.1080/03069885.2012.700506

[24] Hobfoll, S. E. (1989). Conservation of resources: A new attempt at conceptualizing stress. American Psychologist, 44(3), 513–524. DOI: 10.1037/0003-066X.44.3.513 2648906

[25] Hobfoll, S. E. (1998). Stress, culture, and community: The psychology and philosophy of stress. New York, NY: Plenum. DOI: 10.1007/978-1-4899-0115-6

[26] Hobfoll, S. E. (2002). Social and psychological resources and adaptation. Review of General Psychology, 6(4), 307–324. DOI: 10.1037/1089-2680.6.4.307

[27] Hobfoll, S. E. (2011). Conservation of resource caravans and engaged settings. Journal of Occupational and Organizational Psychology, 84(1), 116–122. DOI: 10.1111/j.2044-8325.2010.02016.x

[28] Hobfoll, S. E., & Shirom, A. (2000). Conservation of resources theory: Applications to stress and management in the workplace. In R. T. Golembiewski (Ed.), Handbook of Organizational Behavior (2nd revised edition; pp. 57–81). New York: Marcel Dekker.

[29] Hu, J., & Liden, R. C. (2011). Antecedents of team potency and team effectiveness: An examination of goal and process clarity and servant leadership. Journal of Applied Psychology, 96(4), 851–862. DOI: 10.1037/a0022465 21319877

[30] Judge, T. A., Zhang, S. C., & Glerum, D. R. (2020). Job satisfaction. In Essentials of job attitudes and other workplace psychological constructs, (pp. 207–241). London: Routledge. DOI: 10.4324/9780429325755-11

[31] Kauffeld, S., & Spurk, D. (2022). Why does psychological capital foster subjective and objective career success? The mediating role of career-specific resources. Journal of Career Assessment, 30(2), 285–308. DOI: 10.1177/10690727211040053

[32] Kline, R. (2011). Principles and practice of structural equation modeling (3rd ed). New York: Guilford Press.

[33] Little, T. (2013). Longitudinal Structural Equation Modeling. New York: Guilford Press.

[34] Locke, E. A. (1976). The nature and causes of job satisfaction. In Dunnette, M. D. (Ed.), Handbook of Industrial and Organizational Psychology (pp. 1297–1349). Chicago: Rand-McNally.

[35] Loghman, S., Quinn, M., Dawkins, S., Woods, M., Om Sharma, S., & Scott, J. (2023). A comprehensive meta-analyses of the nomological network of psychological capital (PsyCap). Journal of Leadership & Organizational Studies, 30(1), 108–128. DOI: 10.1177/15480518221107998

[36] Lupșa, D., Vîrga, D., Maricuțoiu, L. P., & Rusu, A. (2020). Increasing psychological capital: A pre-registered meta-analysis of controlled interventions. Applied Psychology, 69(4), 1506–1556. DOI: 10.1016/j.paid.2019.109644

[37] Luthans, F., Avey, J. B., Avolio, B. J., & Peterson, S. J. (2010). The development and resulting performance impact of positive psychological capital. Human Resource Development Quarterly, 21(1), 41–67. DOI: 10.1002/hrdq.20034

[38] Luthans, F., Avolio, B. J., Walumbwa, F. O., & Li, W. (2005). The psychological capital of Chinese workers: Exploring the relationship with performance. Management and Organization Review, 1(2), 249–271. DOI: 10.1111/j.1740-8784.2005.00011.x

[39] Luthans, F., Youssef, C. M., & Avolio, B. J. (2007). Psychological capital. New York, NY: Oxford University press. DOI: 10.1037/t06483-000

[40] MacKinnon, D. P., Lockwood, C. M., & Williams, J. (2004). Confidence limits for the indirect effect: distribution of the product and resampling methods. Multivariate Behavioral Research, 39(1), 99–128. DOI: 10.1207/s15327906mbr3901_4 20157642 PMC2821115

[41] McCallum, S. Y., Forret, M. L., & Wolff, H. G. (2014). Internal and external networking behavior: An investigation of relationships with affective, continuance, and normative commitment. Career Development International, 19(5), 594–614. DOI: 10.1108/CDI-08-2013-0101

[42] Michael, J., & Yukl, G. (1993). Managerial level and subunit function as determinants of networking behavior in organizations. Group & Organization Management, 18(3), 328–351. DOI: 10.1177/1059601193183005

[43] Muthén, L. K., & Muthén, B. O. (2010). Mplus user’s guide: statistical analysis with latent variables: User’s guide. Muthén & Muthén.

[44] Newman, A., Ucbasaran, D., Zhu, F. E. I., & Hirst, G. (2014). Psychological capital: A review and synthesis. Journal of Organizational Behavior, 35(S1), 120–138. DOI: 10.1002/job.1916

[45] Nigah, N., Davis, A. J., & Hurrell, S. A. (2012). The impact of buddying on psychological capital and work engagement: An empirical study of socialization in the professional services sector. Thunderbird International Business Review, 54(6), 891–905. DOI: 10.1002/tie.21510

[46] Nolzen, N. (2018). The concept of psychological capital: A comprehensive review. Management Review Quarterly, 68(3), 237–277. DOI: 10.1007/s11301-018-0138-6

[47] Saks, A. M., & Gruman, J. A. (2011). Organizational socialization and positive organizational behaviour: Implications for theory, research, and practice. Canadian Journal of Administrative Sciences, 28(1), 14–26. DOI: 10.1002/cjas.169

[48] Schaufeli, W. B., Bakker, A. B., & Salanova, M. (2006). The measurement of work engagement with a short questionnaire a cross-national study. Educational and Psychological Measurement, 66(4), 701–716. DOI: 10.1177/0013164405282471

[49] Seligman, M. E., & Csikszentmihalyi, M. (2000). Positive psychology: An introduction. American Psychologist, 55(1), 5–14. DOI: 10.1037/0003-066X.55.1.5 11392865

[50] Simons, J. C., & Buitendach, J. H. (2013). Psychological capital, work engagement and organisational commitment amongst call centre employees in South Africa. SA Journal of Industrial Psychology, 39(2), 1–12. DOI: 10.4102/sajip.v39i2.1071

[51] Paek, S., Schuckert, M., Kim, T. T., & Lee, G. (2015). Why is hospitality employees’ psychological capital important? The effects of psychological capital on work engagement and employee morale. International Journal of Hospitality Management, 50, 9–26. DOI: 10.1016/j.ijhm.2015.07.001

[52] Pena, L., Curado, C., & Oliveira, M. (2022). The contribution of LinkedIn use to career outcome expectations. Journal of Business Research, 144, 788–796. DOI: 10.1016/j.jbusres.2021.09.047

[53] Podsakoff, P. M., MacKenzie, S. B., & Podsakoff, N. P. (2012). Sources of method bias in social science research and recommendations on how to control it. Annual Review of Psychology, 65, 539–569. DOI: 10.1146/annurev-psych-120710-100452 21838546

[54] Porter, C. M., & Woo, S. E. (2015). Untangling the networking phenomenon: A dynamic psychological perspective on how and why people network. Journal of Management, 41(5), 1477–1500. DOI: 10.1177/0149206315582247

[55] Porter, C. M., Woo, S. E., & Campion, M. A. (2016). Internal and external networking differentially predict turnover through job embeddedness and job offers. Personnel Psychology, 69(3), 635–672. DOI: 10.1111/peps.12121

[56] Preacher, K. J., & Hayes, A. F. (2008). Asymptotic and resampling strategies for assessing and comparing indirect effects in multiple mediator models. Behavior Research Methods, 40(3), 879–891. DOI: 10.3758/BRM.40.3.879 18697684

[57] Santilli, S., Marcionetti, J., Rochat, S., Rossier, J., & Nota, L. (2016). Career adaptability, hope, optimism, and life satisfaction in Italian and Swiss adolescents. Journal of Career Development, 44(1), 62–76. DOI: 10.1177/0894845316633793

[58] ten Brummelhuis, L. L., & Bakker, A. B. (2012). A resource perspective on the work–home interface: The work–home resources model. American Psychologist, 67(7), 545–556. DOI: 10.1037/a0027974 22506688

[59] Thoits, P. A. (1994). Stressors and problem-solving: The individual as psychological activist. Journal of Health and Social Behavior, 35(2), 143–160. DOI: 10.2307/2137362 8064122

[60] Tras, Z., Sunbul, M. G., & Baltaci, U. B. (2021). Investigation of the relationships between optimism, perceived social support, and hope. ie: inquiry in education, 13(1), 11.

[61] Vogt, K., Hakanen, J. J., Brauchli, R., Jenny, G. J., & Bauer, G. F. (2016). The consequences of job crafting: A three-wave study. European Journal of Work and Organizational Psychology, 25(3), 353–362. DOI: 10.1080/1359432X.2015.1072170

[62] Volmer, J., Schulte, E. M., Handke, L., Rodenbücher, L., & Tröger, L. (2021). Do all employees benefit from daily networking? The moderating effect of the affiliation motive. Journal of Career Development, 48(5), 555–568. DOI: 10.1177/0894845319873727

[63] Volmer, J., & Wolff, H. G. (2018). A daily diary study on the consequences of networking on employees’ career-related outcomes: The mediating role of positive affect. Frontiers in Psychology, 9, 2179. DOI: 10.3389/fpsyg.2018.02179 30483188 PMC6243093

[64] Wang, H., & Lei, L. (2023). Proactive personality and job satisfaction: Social support and hope as mediators. Current Psychology, 42(1), 126–135. DOI: 10.1007/s12144-021-01379-2

[65] Wingender, L. M., & Wolff, H. G. (2023). The dark and bright side of networking behavior: Three studies on short-term processes of networking behavior. Journal of Vocational Behavior, 140, 103811. DOI: 10.1016/j.jvb.2022.103811

[66] Wolff, H. G. & Moser, K. (2006). Entwicklung und Validierung einer Networkingskala (Development and validation of a networking scale). Diagnostica, 52(4), 161–80. DOI: 10.1026/0012-1924.52.4.161

[67] Wolff, H. G., & Moser, K. (2009). Effects of Networking on Career Success: A Longitudinal Study. Journal of Applied Psychology, 94(1), 196–206. DOI: 10.1037/a0013350 19186904

[68] Wolff, H. G., Moser, K., & Grau, A. (2008). Networking: Theoretical foundations and construct validity. In J. Deller (Ed.), Readings in applied organizational behavior from the Lüneburg symposium – Personality at work (pp. 101–118). Mering: Rainer Hampp.

[69] Wolff, H. G., Schneider-Rahm, C. I., & Forret, M. L. (2011). Adaptation of a German multidimensional networking scale into English. European Journal of Psychological Assessment, 27(4), 244–250. DOI: 10.1027/1015-5759/a000070

[70] Wolff, H. G., Weikamp, J. G., & Batinic, B. (2018). Implicit motives as determinants of networking behaviors. Frontiers in Psychology, 9, 411. DOI: 10.3389/fpsyg.2018.00411 29760668 PMC5936778

[71] Wolter, C., Santa Maria, A., Gusy, B., Lesener, T., Kleiber, D., & Renneberg, B. (2019). Social support and work engagement in police work: The mediating role of work–privacy conflict and self-efficacy. Policing: An International Journal, 42(6), 1022–1037. DOI: 10.1108/PIJPSM-10-2018-0154

[72] Wu, W. Y., & Nguyen, K.-V. H. (2019). The antecedents and consequences of psychological capital: a meta-analytic approach. Leadership and Organization Development Journal, 40(4), 435–456. DOI: 10.1108/LODJ-06-2018-0233

